# Supplementary material for: Kin discrimination promotes horizontal gene transfer between unrelated strains in Bacillus subtilis
Source: Nat Commun. 2021 Jun 8;12:3457. doi: 10.1038/s41467-021-23685-w (PMC8187645; doi:10.1038/s41467-021-23685-w)
Supplement: Supplementary file 3 — Reporting Summary [file 41467_2021_23685_MOESM3_ESM.pdf]

nature research

Corresponding author(s): Stefanic Polonca and Ines Mandic Mulec

Last updated by author(s): Apr 21, 2021

Reporting Summary

Nature Research wishes to improve the reproducibility of the work that we publish. This form provides structure for consistency and transparency in reporting. For further information on Nature Research policies, see our Editorial Policies and the Editorial Policy Checklist.

Statistics

For all statistical analyses, confirm that the following items are present in the figure legend, table legend, main text, or Methods section.

n/a	Confirmed	
[ ]	[x]	The exact sample size (*n*) for each experimental group/condition, given as a discrete number and unit of measurement
[ ]	[x]	A statement on whether measurements were taken from distinct samples or whether the same sample was measured repeatedly
[ ]	[x]	The statistical test(s) used AND whether they are one- or two-sided<br>*Only common tests should be described solely by name; describe more complex techniques in the Methods section.*
[x]	[ ]	A description of all covariates tested
[x]	[ ]	A description of any assumptions or corrections, such as tests of normality and adjustment for multiple comparisons
[ ]	[x]	A full description of the statistical parameters including central tendency (e.g. means) or other basic estimates (e.g. regression coefficient) AND variation (e.g. standard deviation) or associated estimates of uncertainty (e.g. confidence intervals)
[ ]	[x]	For null hypothesis testing, the test statistic (e.g. *F*, *t*, *r*) with confidence intervals, effect sizes, degrees of freedom and *P* value noted<br>*Give P values as exact values whenever suitable.*
[x]	[ ]	For Bayesian analysis, information on the choice of priors and Markov chain Monte Carlo settings
[x]	[ ]	For hierarchical and complex designs, identification of the appropriate level for tests and full reporting of outcomes
[x]	[ ]	Estimates of effect sizes (e.g. Cohen's *d*, Pearson's *r*), indicating how they were calculated

Our web collection on statistics for biologists contains articles on many of the points above.

Software and code

Policy information about availability of computer code

Data collection	No newly developed code was used in the data collection step. Genome sequences were obtained using CLC Genomics Workbench 9 assembler using default parameters.
Data analysis	Genome ANI was computed using FASTani 0.1.2 (https://github.com/kbaseapps/FastANI). Pictures were analysed with Fiji 1.51d, No custom code were used. Mean, standard deviation and T-test calculations were performed using Microsoft Excel 12.00.

For manuscripts utilizing custom algorithms or software that are central to the research but not yet described in published literature, software must be made available to editors and reviewers. We strongly encourage code deposition in a community repository (e.g. GitHub). See the Nature Research guidelines for submitting code & software for further information.

Data

Policy information about availability of data

All manuscripts must include a data availability statement. This statement should provide the following information, where applicable:

- Accession codes, unique identifiers, or web links for publicly available datasets
- A list of figures that have associated raw data
- A description of any restrictions on data availability

The data that support the findings of this study are available from the corresponding author upon reasonable request and in Source Data file. Genome sequences are available in the NCBI database under genome accession numbers VBRL00000000, VBRM00000000, VBRN00000000, VBRO00000000, VBRQ00000000 and VBRR00000000.
This data availability statement is included in the manuscript.

Field-specific reporting

Please select the one below that is the best fit for your research. If you are not sure, read the appropriate sections before making your selection.

- [x] Life sciences
- [ ] Behavioural & social sciences
- [ ] Ecological, evolutionary & environmental sciences

For a reference copy of the document with all sections, see nature.com/documents/nr-reporting-summary-flat.pdf

Life sciences study design

All studies must disclose on these points even when the disclosure is negative.

Sample size	No statistical tests were used to determine sample sizes. Such was chosen based on experience and knowledge on expected biological variability (which cannot be controlled by the experimenter) and accuracy of the experimental methods used. we used 3 independent biological experiment that were performed in 3 technical replicates. 3 independent experiments are very common in biological samples (DOI: 10.1021/acssynbio.9b00075; 10.1371/journal.pone.0238993; 10.1371/journal.pgen.1006474; 10.1016/j.cub.2016.01.032; 10.1111/1751-7915.12258; 10.1186/s12934-015-0341-2; 10.3390/microorganisms8081131)
Data exclusions	No data were excluded from the analyses.
Replication	All attempts at experiments replications were successful. Experiments were performed 3 times in independent biological experiments in 3 technical replicates.
Randomization	Randomization was not relevant in this study. Comparisons were not performed on individual organisms (with high level of individual variability) but on large populations of microbes that were independently initiated from small number of cells. Growth conditions were strictly controlled by the experimenter and identical for each population.
Blinding	Blinding was not relevant in this study, because methods used in the assessment of the results were objective. Blinding is also not necessary because the results are quantitative and did not require subjective judgment or interpretation. Blinding is not typically used in the field.

Reporting for specific materials, systems and methods

We require information from authors about some types of materials, experimental systems and methods used in many studies. Here, indicate whether each material, system or method listed is relevant to your study. If you are not sure if a list item applies to your research, read the appropriate section before selecting a response.

Materials & experimental systems

n/a	Involved in the study	
x		Antibodies
x		Eukaryotic cell lines
x		Palaeontology and archaeology
x		Animals and other organisms
x		Human research participants
x		Clinical data
x		Dual use research of concern

Methods

n/a	Involved in the study	
x		ChIP-seq
x		Flow cytometry
x		MRI-based neuroimaging
